# Developing a deep learning model for predicting ovarian cancer in Ovarian-Adnexal Reporting and Data System Ultrasound (O-RADS US) Category 4 lesions: A multicenter study

**DOI:** 10.1007/s00432-024-05872-6

**Published:** 2024-07-09

**Authors:** Wenting Xie, Wenjie Lin, Ping Li, Hongwei Lai, Zhilan Wang, Peizhong Liu, Yijun Huang, Yao Liu, Lina Tang, Guorong Lyu

**Affiliations:** 1 Department of Ultrasound Medicine, The Second Affiliated Hospital of Fujian medical University, Quanzhou, Fujian Province 362000 China; 2 Department of Ultrasound, Fujian Cancer Hospital, Clinical Oncology School of Fujian Medical University, Fuzhou, Fujian Province 350014 China; 3 Quanzhou Bolang Technology Group Co., Ltd, Quanzhou, Fujian Province 362000 China; 4 Department of Gynecology and Obstetrics, Quanzhou First Hospital Affiliated to Fujian Medical University, Quanzhou, Fujian 362000 China; 5 Department of Ultrasound, Fujian Provincial Maternity and Children’s Hospital, Fuzhou, Fujian Province 350014 China; 6 Department of Ultrasound, Nanping First Hospital Affiliated to Fujian Medical University, Nanping, Fujian Province 35300 China; 7 School of Medicine, Huaqiao University, Quanzhou, Fujian Province 362000 China

**Keywords:** Ovarian cancer, Deep learning, Ultrasonography, Ovarian-adnexal reporting and data system

## Abstract

**Purpose:**

To develop a deep learning (DL) model for differentiating between benign and malignant ovarian tumors of Ovarian-Adnexal Reporting and Data System Ultrasound (O-RADS US) Category 4 lesions, and validate its diagnostic performance.

**Methods:**

A retrospective analysis of 1619 US images obtained from three centers from December 2014 to March 2023. DeepLabV3 and YOLOv8 were jointly used to segment, classify, and detect ovarian tumors. Precision and recall and area under the receiver operating characteristic curve (AUC) were employed to assess the model performance.

**Results:**

A total of 519 patients (including 269 benign and 250 malignant masses) were enrolled in the study. The number of women included in the training, validation, and test cohorts was 426, 46, and 47, respectively. The detection models exhibited an average precision of 98.68% (95% CI: 0.95–0.99) for benign masses and 96.23% (95% CI: 0.92–0.98) for malignant masses. Moreover, in the training set, the AUC was 0.96 (95% CI: 0.94–0.97), whereas in the validation set, the AUC was 0.93(95% CI: 0.89–0.94) and 0.95 (95% CI: 0.91–0.96) in the test set. The sensitivity, specificity, accuracy, positive predictive value, and negative predictive values for the training set were 0.943,0.957,0.951,0.966, and 0.936, respectively, whereas those for the validation set were 0.905,0.935, 0.935,0.919, and 0.931, respectively. In addition, the sensitivity, specificity, accuracy, positive predictive value, and negative predictive value for the test set were 0.925, 0.955, 0.941, 0.956, and 0.927, respectively.

**Conclusion:**

The constructed DL model exhibited high diagnostic performance in distinguishing benign and malignant ovarian tumors in O-RADS US category 4 lesions.

## Introduction

Ovarian cancer is one of the most common gynecological malignancy and the fifth leading cause of cancer-related deaths in women worldwide (Siegel et al. 2022; Zheng et al. 2020). The cancer lacks typical symptoms which makes it difficult to conduct early screening and timely diagnosis. Consequently, most patients with ovarian cancer are diagnosed at an advanced stage (Terp et al. 2023). Advanced ovarian cancer patients are often treated with debulking surgery combined with platinum and paclitaxel chemotherapy, but still these treatment modalities are associated with poor survival and high recurrence rates (Konstantinopoulos and Matulonis 2023; Wheeler et al. 2023). Currently, the main diagnostic method for ovarian-adnexal lesions is pelvic imaging (Sadowski et al. 2023). Ultrasonography (US) is the most commonly used imaging modality for assessing ovarian-adnexal lesions owing to its universality, non-invasive nature, and affordability (Wang et al. 2021). However, given its pathological diversity and morphological complexity, accurate preoperative diagnosis of ovarian-adnexal masses by conventional US alone has been suboptimal (Shi et al. 2023). To date, several US-based models have been developed to assess the benign or malignant adnexal masses, such as the International Ovarian Tumor Analysis (IOTA) Simple Rules, the Assessment of Different Neoplasia in the Adnexa (ADNEX) model, and Ovarian-Adnexal Reporting and Data System (O-RADS) (Dang Thi Minh et al. 2024; Pelayo et al. 2023; Pozzati et al. 2023).

In 2020, the American College of Radiology proposed the O-RADS risk stratification and management system which provides a detailed description of each category (Andreotti et al. 2018, 2020; Yang et al. 2023). The system recommends six risk classification categories. These include: O-RADS category 0, defined as an incomplete evaluation; O-RADS category 1, defined as the physiologic category; O-RADS category 2, defined as the almost certainly benign category with <1% malignant probability; O-RADS category 3, referring to lesions with 1% to<10% risk of malignancy; O-RADS category 4, defined as lesions with 10% to<50% risk of malignancy and O-RADS category 5, referring to lesions with high risk of malignancy (≥ 50%) (Vara et al. 2022). The accuracy of gynecologic ultrasonography is largely dependent on the sonologist’s subjective assessment. It has been observed that the correct classification of the adnexal lesions in expert ultrasound examination is higher than in less experienced doctors (Wu et al. 2023). Currently, there are no effective management strategies for O-RADS US 4 lesions, with the risk of malignancy exhibiting significant variations and some lesions found to be benign. If benign lesions can be accurately diagnosed, patients can avoid unnecessary or extensive surgery. A simple description of the ovarian tumor by a sonologist may not be completely fulfilled. This calls for the establishment of appropriate and advanced approaches for sub-stratifying O-RADS US 4 lesions into benign and malignant subgroups.

Artificial intelligence (AI) has emerged as a significant tool with diverse medical applications (Wang et al. 2024). Deep learning (DL) can quantitatively analyze of medical images and has been applied in the field of oncology (Taddese et al. 2024). Several studies demonstrated that DL can improve the diagnosis, predict treatment responses, and progression-free survival of patients with ovarian tumors (Arezzo et al. 2022; Boehm et al. 2022; Na et al. 2024; Sadeghi et al. 2024; Yao et al. 2021). Compared with traditional imaging diagnosis by radiologists, the DL method can improve the accuracy and reduce the bias of diagnosis results (Chen et al. 2022). However, few studies have demonstrated the diagnostic performance of AI in O-RADS 4 lesions.

In this multicenter study, we developed and tested a US image-based DL model for distinguishing between benign and malignant ovarian tumors of O-RADS 4 lesions and to demonstrate the diagnostic performance of our DL model.

## Materials and methods

### Study population and datasets

**Fig. 1:**
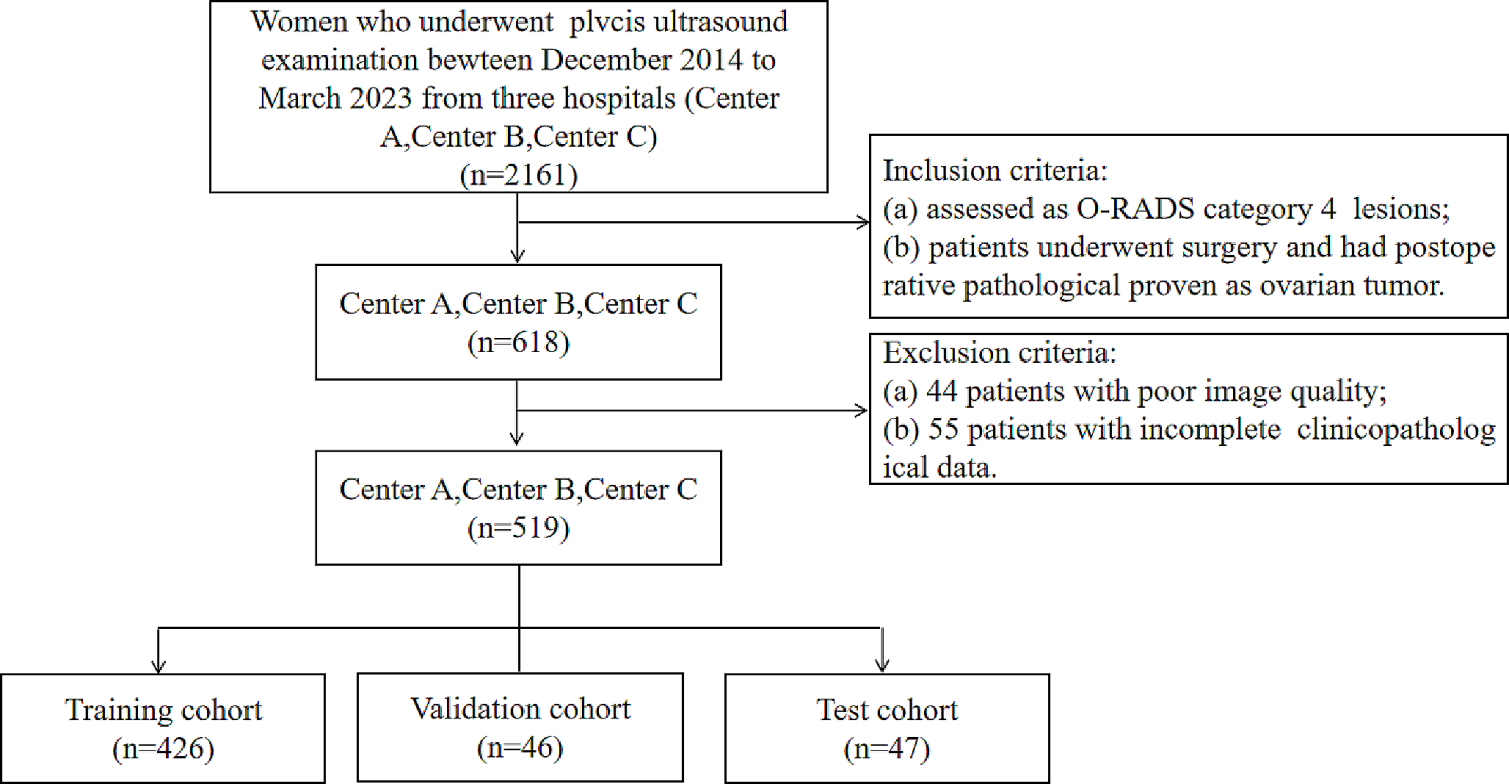
The flowchart of patient enrollment, inclusion, and exclusion criteria, and partitioning of datasets. Center A, the second affiliated hospital of Fujian Medical University; Center B, Fujian Cancer Hospital; Center C, Nanping First Hospital Affiliated to Fujian Medical University

This retrospective study was approved by the Institutional Review Board of the Second Affiliated Hospital of Fujian Medical University (No.636, 2023). The analyzed datasets were obtained from three hospitals from December 2014 to March 2023. The three centers were coded as A, the Second Affiliated Hospital of Fujian Medical University; B, Fujian Cancer Hospital; and C, Nanping First Hospital Affiliated to Fujian Medical University. Consecutive patients who met the following criteria were included: (1) underwent diagnostic pelvis US prior to gynecological surgery; (2) diagnosed with O-RADS category 4 by US radiologists according to the O-RADS lexicon white paper; (3) postoperative pathological confirmation of ovarian tumor; (4) did not use radiotherapy or chemotherapy before US examination. Patients with poor image quality and without histopathology were excluded. A total of 519 women met the inclusion criteria and were enrolled in this study (Fig. 1). Center C was used as the validation set, 10% of all cases coded Center A and Center B as the test set, and the remaining 90% as the training set.

The US image target masses with the largest diameter or a more complicated ultrasound morphology were selected for further analysis. The US images were acquired using ultrasound devices equipped with abdominal probes spanning frequencies from 1 to 6 MHz, as well as transvaginal probes covering frequencies from 2 to 9 MHz. Confirmation of all adnexal lesions was achieved by surgical pathology. Furthermore, pertinent clinical data such as age at diagnosis, lesion size, CA125 levels, and menopausal status were meticulously documented for subsequent analysis.

### Algorithm for analysis

#### Image annotation

First, we used a professional image annotation tool LabelImg (https://github.com/HumanSignal/labelImg) to draw bounding boxes on all ovarian cancer images to mark the location and extent of ovarian tumors. The boundaries of each lesion area were drawn by two radiologists (with 6 and 3 years of experience). The boundaries of all lesions were manually delineated on the axes of the 2D images. In addition, we accurately outlined the tumor borders, ensuring precise adjustment of the bounding box to accommodate the morphology of the tumor. In addition to the bounding box labeling, each bounding box was assigned a corresponding category label, benign or malignant.

#### Feature extraction

The dataset was preprocessed which involved processed such as image specification and data enhancement. Images were then resized to the same resolution. In addition, we randomly employed data enhancement techniques such as flipping, cropping, and addition of noise to the images to generate richer training data to improve the generalization of the model. To normalize the range of values for each phenotypic feature, the feature normalization technique was employed to convert these features to 0 or 1. Next, segmentation feature extraction was conducted using DeepLabV3 (Chen et al. 2017). DeepLabV3 utilizes the architecture of domain-adaptive convolutional neural networks to capture the regional features of objects. Within the encoder-decoder architecture, the encoder module employs ResNet-101 (Jusman, 2023) as its backbone network for extracting low-level features. Subsequently, the decoder module utilizes null convolution to augment edge features, followed by up-sampling to reconstruct the segmentation output. In addition, we used the YOLOv8 (Terven et al. 2023) network to extract the target detection features. YOLOv8 adopts Darknet-53 (Azim et al., 2022) as the backbone network to improve the inter-channel correlation of the feature channels while preserving the fine edge information due to the new channel attention module and short connections. The output feature maps were post-processed to predict the edge coordinates and the classification.

### Machine learning model development

**Fig. 2:**
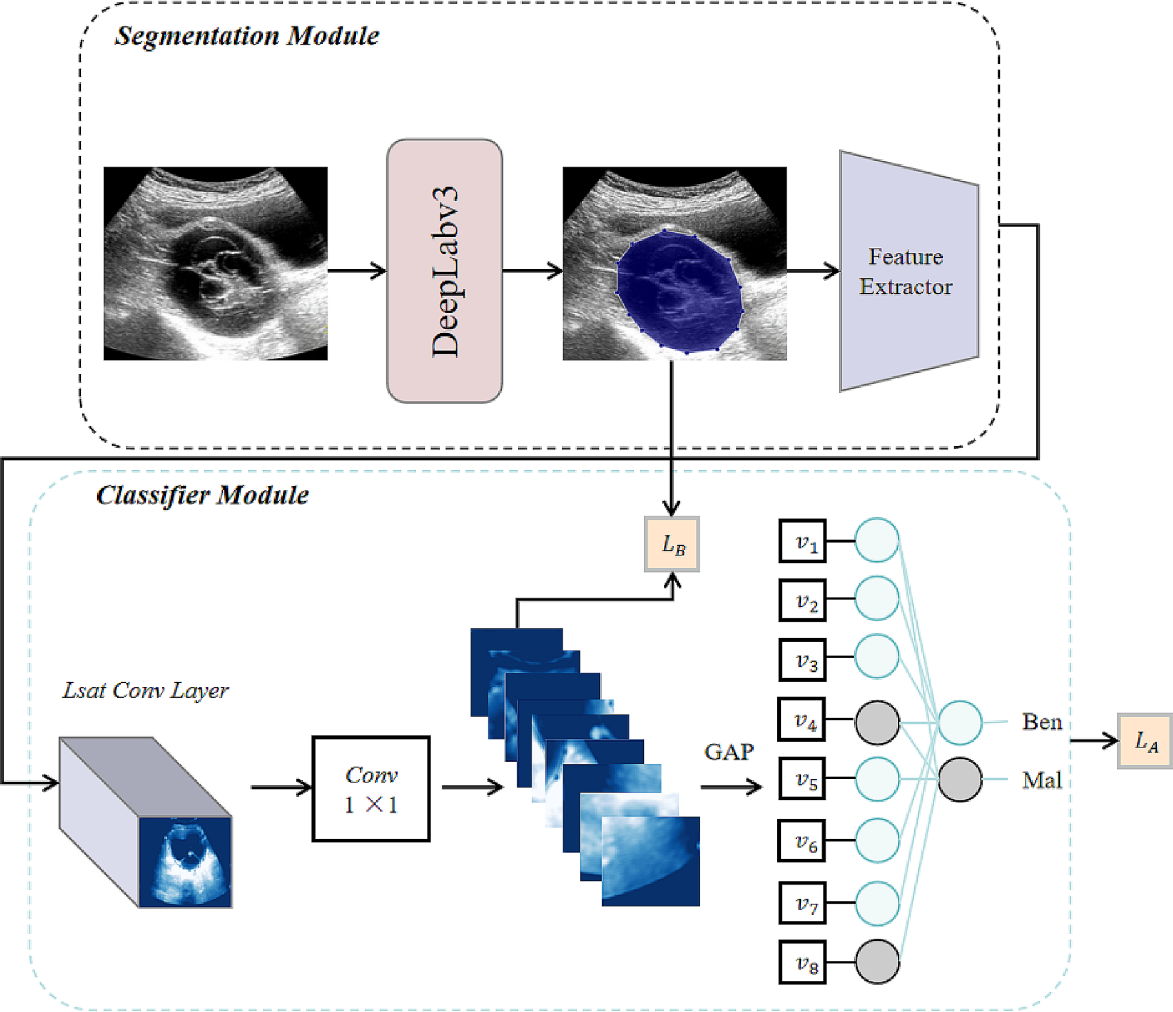
The overview of the proposed framework. Our approach is divided into two major phases: (1) The segmentation stage in which the DeepLabV3 model is used to obtain the lesion segmentation mask, which is subsequently used to mask the input image. (2) In the classifier phase, YOLOv8 is employed to disentangle the feature maps of a CNN into human-interpretable concepts

Furthermore, we propose a novel dual-model architecture (Fig. 2) that leverages the complementary abilities of DeepLabV3 and YOLOv8. In our approach, we employed DeepLabV3 to first perform pixel-accurate segmentation of ovarian tumor regions from US images. The ResNet-101pretrained on ImageNet was employed as the encoder backbone in DeepLabV3. Atrous separable convolutions were utilized with varying rates to explicitly capture multi-scale information. Moreover, a lightweight decoder module was integrated to restore fine spatial details and generate segmentation maps at the full image resolution. These segmented outputs were subsequently fed into YOLOv8 for downstream classification and detection tasks. Notably, YOLOv8 features a modified Darknet-53 network, which consists of residual and convolutional blocks, serving as the backbone for feature extraction. YOLOv8 classifies each tumor region as benign or malignant and generates bounding boxes around individual tumor objects. This enables joint analysis of segmentation, classification, and localization within a single framework. To achieve better outcomes, we first trained DeepLabV3 on our US image datasets until convergence was attained. The model outputs were then extracted and fed to YOLOv8 in the training phase. Both models were fine-tuned end-to-end through this sequential process. At inference, a new US image is directly input to obtain segmented tumor regions, which are instantly classified and localized by YOLOv8.

### Model training

A pipeline was established to extract tumor ROIs at 512 × 512 pixels from the annotated masks while maintaining original aspect ratios. To address the class imbalance, we oversampled the minority malignant class during the training stage. We trained DeepLabV3 and YOLOv8 sequentially in an end-to-end fashion on our dataset through transfer learning. DeepLabV3 was first pretrained on PASCAL VOC3 to achieve semantic segmentation. Subsequently, we fine-tuned the model on our ovarian tumor masks for 50 epochs. This process employed stochastic gradient descent with polynomial learning rate decay, utilizing a batch size of 4 and employing the dice coefficient loss function. After segmentation, YOLOv8 was initialized from weights pretrained on COCO4 for object detection. It was then optimized on the extracted DeepLabV3 tumor regions for 100 epochs. The proposed dual-model architecture and training procedure presents an innovative approach for joint analysis of US images.

### Statistical analysis

All statistical analyses were conducted using the SPSS 20.0 software (IBM, Armonk, NY, USA). Categorical data were analyzed by Chi-Squared test and expressed as the frequency and percentage. Continuous data were analyzed by the Student’s *t* test or Mann-Whitney *U* test and then expressed as the mean and standard deviation or median and interquartile range. The ROC curve analysis was utilized to evaluate the performance of our proposed dual-model method. The ROC curves were constructed by the ROC package on Python (version 3.8.0). *p* < 0.05 was considered statistically significant.

## Results

### Patient characteristics

A total of 1619 images in 519 patients who underwent US examination and surgery were enrolled from three hospitals. The histological profile of enrolled adnexal patients is presented in Table 1. The number of women included in the training, validation, and test cohorts was 426, 46, and 47, respectively.

**Table 1:** Pathology results of 519 adnexal masses that were assigned O-RADS US category 4

Center A (*n* = 156)		Center B (*n* = 317)		Center C (*n* = 46)	
Histopathological findings	No. of patients (%)	Histopathological findings	No. of patients (%)	Histopathological findings	No. of patients (%)
**Benign**		**Benign**		**Benign**	
Benign cyst	5(3.2)	Benign cyst	13(4.1)	Serous cyst	2(4.3)
Endometriosis cyst	6(3.8)	Endometriosis cyst	21(6.6)	Endometriosis cyst	6(13.0)
Teratoma	8(5.1)	Teratoma	17(5.4)	Teratoma	5(10.9)
Cystadenoma	24(15.4)	Cystadenofibroma	7(2.2)	Cystadenoma	22(47.8)
Struma ovarii	1(0.6)	Cystadenoma	61(19.2)	Struma ovarii	1(2.2)
Inflammation	5(3.2)	Struma ovarii	10(3.2)	Fibroma	1(2.2)
Cystadenofibroma	4(2.6)	Brenner	2(0.6)	Sclerosing stromal tumor	1(2.2)
Fibroma	6(3.8)	Inflammation	5(1.6)		
Theca-fibroma	1(0.6)	Fibroma	4(1.3)		
		Theca-fibroma	30(9.5)		
		Microcystic stromal tumour	1(0.3)		
**Malignant**		**Malignant**		**Malignant**	
Borderline	46(29.5)	Borderline	71(22.4)	Borderline	6(13.0)
High grade	33(21.2)	High grade	32(10.1)	High grade	1(2.2)
Immature teratoma	5(3.2)	Low grade	2(0.6)	Metastasis	1(2.2)
Clear cell carcinoma	5(3.2)	Immature teratoma	3(1.0)		
Endometrioid cancer	3(2.0)	Clear cell carcinoma	14(4.4)		
Granular cell tumor	3(2.0)	Endometrioid cancer	5(1.6)		
Sertoli-Leydig cell tumor	1(0.6)	Granular cell tumor	9(2.8)		
		Sertoli-Leydig cell tumor	1(0.3)		
		Metastasis	8(2.5)		
		Malignant mixed Mullerian tumour	1(0.3)		

Center A, the second affiliated hospital of fujian medical university; Center B, Fujian Cancer Hospital; Center C, Nanping First Hospital Affiliated to Fujian Medical University

The clinical characteristics and laboratory results of patients are shown in Table 2. It can be inferred that 269 (51.8%) lesions were benign while 250 (48.2%) lesions were malignant. Among the study variables, the largest diameter of the lesions (mm) (108 (75.5-142.6) vs. 120.5 (77.7-171.7) mm, *p* = 0.024) was significantly different between benign and malignant groups. In women with elevated CA125 levels, malignant tumors were significantly more prevalent than benign tumors (*p* < 0.001). Nevertheless, no statistically significant variances were detected in terms of age at diagnosis, menopausal status, or tumor location between the benign and malignant groups.

**Table 2:** Comparison clinical features between benign and malignant O-RADS 4 US adnexal lesions

Characteristic	Benign (*n* = 269)	Malignant (*n* = 250)	*p*-value
Age at diagnosis	47.0 ± 15.9	47.2 ± 14.5	0.866
Largest diameter of lesion (mm)	108(75.5−142.6)	120.5(77.7−171.7)	0.024
Menopausal status			0.631
Premenopausal	145(53.9)	140(56.0)	
Postmenopausal	124(46.1)	110(44.0)	
Location			0.38
Left	126(46.8)	118(47.2)	
Right	119(44.2)	101(40.4)	
Bilateral	24(9.0)	31(12.4)	
Serum CA125 level (U/ml)			0.000
≤ 35	176(65.4)	105(42.0)	
>35	93(34.6)	145(58.0)	

### Model performance

**Fig. 3:**
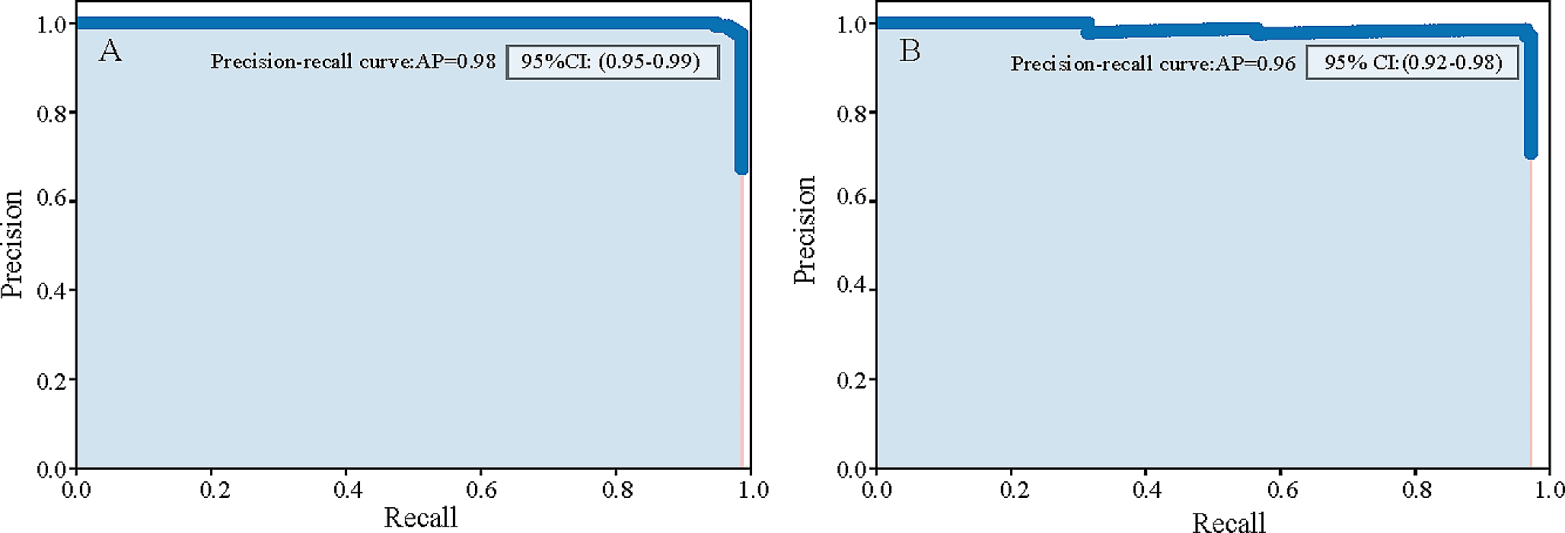
The performance of the adnexal mass detection model. (**A**) The precision-recall curve of the detection model used for evaluating for performance of benign masses indicating an average precision (AP) of 0.98 (95% CI: 0.95–0.99). (**B**) The precision-recall curve of the detection model used for evaluating for performance of malignant masses displaying an AP of 0.96 (95% CI: 0.92–0.98)

**Fig. 4:**
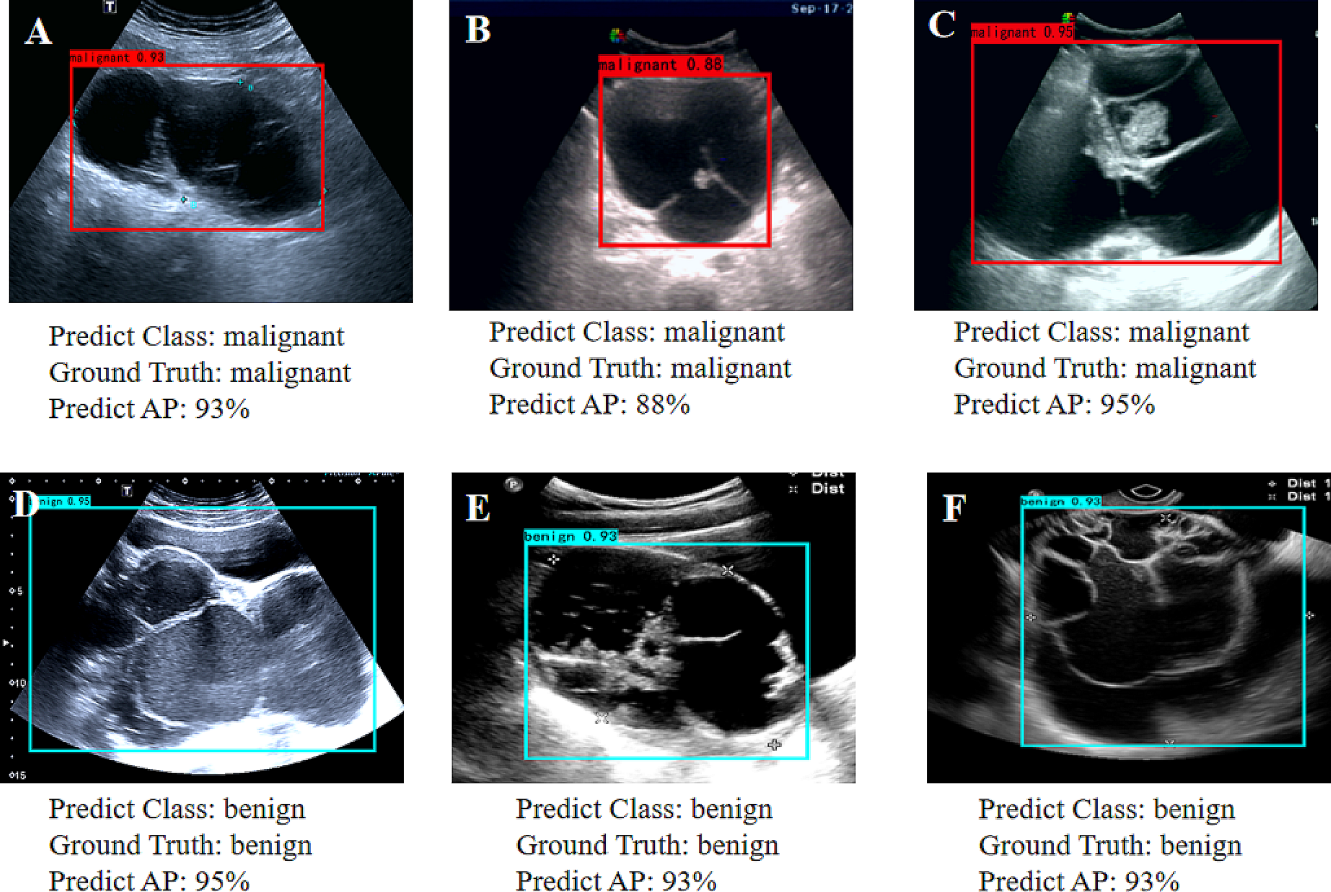
Images with O-RADS US category 4 masses detected using the model. (**A**) High-grade serous carcinoma in a 70-year-old female patient, (**B**) Borderline serous tumor in a 57-year-old female patient, (**C**) Brenner (borderline) in a 69-year-old female patient, (**D**) Endometriosis cyst in a 22-year-old female patient, (**E**) Teratoma in a 22-year-old female patient, (**F**) Mucinous cystadenoma in a 47-year-old female patient. The red and blue bounding box within each imaging indicates the adnex masses delineated by the detection model

The performance of the detection DL model is shown in Fig. 3. The average precision of the precision-recall curve was 98.68% (95% CI: 0.95–0.99) for benign masses (Fig. 3A) and 96.23% (95% CI: 0.92–0.98) for malignant masses (Fig. 3B). When applied to US imaging of adnexal masses, the model showed the potential to identify nodules in benign and malignant categories (Fig. 4).

**Fig. 5:**
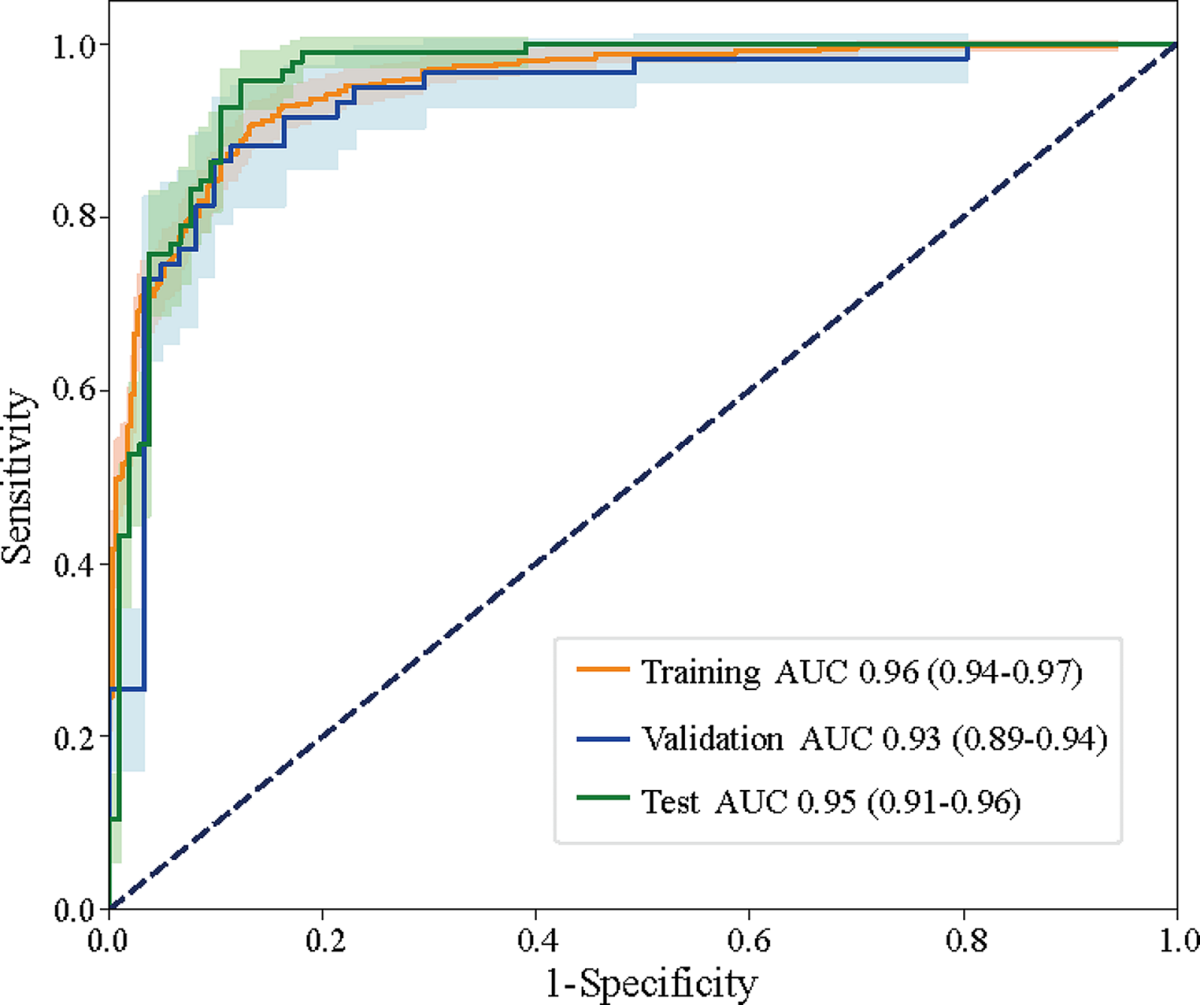
The ROC curve showing the classification model for ovarian cancer classification

The DL model had the best discrimination between the benign and malignant groups, with an AUC of 0.96 (95% CI: 0.94–0.97) in the training set, an AUC of 0.93(95% CI: 0.89–0.94) in the validation set and 0.95 (95% CI: 0.91–0.96) in the test set (Fig. 5). Further analysis indicated that the sensitivity, specificity, accuracy, positive predictive value, and negative predictive value in the training set were 0.943, 0.957, 0.951, 0.966, and 0.936, respectively, whereas those for the validation set were 0.905,0.935, 0.935,0.919, and 0.931, respectively. In addition, the sensitivity, specificity, accuracy, positive predictive value, and negative predictive value for the test set were 0.925,0.955,0.941,0.956, and 0.927, respectively (Table 3).

**Table 3:** Diagnostic performance of the deep learning classification model in training, validation, and test cohorts

Set	AUC	Sensitivity	Specificity	Accuracy	Positive predictive value	Negative predictive value
Training set	0.96(95%CI:0.94–0.97)	0.943	0.957	0.951	0.966	0.936
Validation set	0.93(95%CI:0.89–0.94)	0.905	0.935	0.927	0.919	0.931
Test set	0.95(95%CI:0.91–0.96)	0.925	0.955	0.941	0.956	0.927

## Discussion

The significant similarity in ultrasonographic characteristics between malignant and benign ovarian lesions posed a diagnostic challenge for sonologists. Nevertheless, prior research has demonstrated that O-RADS US can accurately detect ovarian malignancies, indicating outstanding diagnostic accuracy (Hack et al. 2022). However, the risk of malignancy in O-RADS 4 was in the range of 10% to<50% implying that some benign lesions were classified in this category. Correct classification of an adnexal lesion is therefore important for improving personalized management. Xu et al. incorporated the qualitative parameters of contrast-enhanced ultrasound (CEUS) to reassign the O-RADS category and the overall sensitivity increased to 90.2% (Xu et al. 2023). In this study, we evaluated the performance of the DL model to classify benign or malignant lesions in O-RADS US Category 4 lesions and showed an acceptable diagnostic performance with an AUC of 0.95.

We deployed a DL method for distinguishing O-RADS 4 lesions. DeepLabV3 is well-suited for precise semantic segmentation due to its powerful encoder-decoder design. Chen et al. developed DeepLabV3 to facilitate the segmentation of colon cancer histology at subcellular scales (Chen et al. 2018). However, semantic segmentation alone does not provide classification or localization of tumors. Object detection models such as YOLO have achieved great success in natural images. In a previous study, Xiao et al. employed YOLOv3 to identify lung cancer in CT scans (Xiao et al. 2023). Meanwhile, YOLOv8 has been shown to rapidly classify and achieve object detection due to its robust architecture (Redmon 2018). In this study, we leveraged the strengths of these models to conduct an in-depth analysis of US images. Our innovative dual-model architecture merges the features of DeepLabV3 and YOLOv8, enabling concurrent segmentation, classification, and detection of ovarian tumors. DeepLabV3 first segments tumor regions, whose outputs are then classified and localized by YOLOv8. This novel dual-model architecture can enable efficient and accurate analysis of imaging analysis.

Artificial intelligence has been demonstrated to improve the diagnostics rate of ovarian tumors. A study found that using deep neural networks to analyze ultrasound images can discriminate between benign and malignant ovarian masses and achieve comparable diagnostic accuracy to expert examiners (Christiansen et al. 2021). A recent multicenter study developed a deep convolutional neural network (DCNN) model for detecting ovarian cancer with high performance (Gao et al. 2022). By integrating DeepLabV3 and YOLOv8, our DL system achieved a higher AUC. However, we cannot directly compare our results to those obtained in previous studies because our focus was on a specific population subset, and the ovarian tumor datasets utilized differ from those in previous investigations.

In addition, we investigated the clinical features of benign and malignant O-RADS 4 US lesions. The levels of CA-125 were higher in malignant masses than in benign masses (*p*<0.05), which is consistent with recent literature (Wong et al. 2023). Not surprisingly, the largest diameter of the lesion was significantly higher in malignant masses than in benign masses (*p* = 0.024). This finding may be ascribed to the concept that ovarian tumors often lack typical symptoms and hence detected at the advanced stage.

This study has several limitations that should be acknowledged. First, this study was a retrospective investigation which may have inherent biases. To improve the DL model’s reliability, a prospective study should be performed. Second, we did not include clinical factors in the DL model. Third, we only used one DL method to construct the model. In our future work, we plan to explore and compare the performance of various DL methods. Moreover, we did not assess the diagnostic accuracy of our DL model against US-based models, such as the IOTA-ADNEX model. Therefore, it will be imperative to conduct a comparative analysis of diagnostic performance between our model and the IOTA-ADNEX model.

## Conclusions

In conclusion, this study demonstrates that the US image-based DL model may be used as a tool for distinguishing between benign and malignant ovarian tumors of O-RADS 4 lesions.

## Data Availability

No datasets were generated or analysed during the current study.

## Abbreviations

O-RADS: Ovarian-Adnexal Reporting and Data System
US: Ultrasonography
DL: Deep learning
AUC: Area under the receiver operating characteristic curve
